# Coupling Osmotic Efficacy with Biocompatibility in Peritoneal Dialysis: A Stiff Challenge

**DOI:** 10.3390/ijms25063532

**Published:** 2024-03-20

**Authors:** Mario Bonomini, Valentina Masola, Maria Pia Monaco, Vittorio Sirolli, Lorenzo Di Liberato, Tommaso Prosdocimi, Arduino Arduini

**Affiliations:** 1 Nephrology and Dialysis Unit, Department of Medicine, G. D’Annunzio University, Chieti-Pescara, SS. Annunziata Hospital, Via dei Vestini, 66013 Chieti, Italy; mariapia.monaco@asl2abruzzo.it (M.P.M.); vittorio.sirolli@unich.it (V.S.); lorenzo.diliberato@asl2abruzzo.it (L.D.L.); 2 Division of Nephrology and Dialysis, Department of Medicine, University of Verona, Piazzale A. Stefani 1, 37126 Verona, Italy; valentina.masola@unipd.it; 3 Department of Biomedical Sciences, University of Padova, Viale G. Colombo 3, 35121 Padova, Italy; 4 Department of Research and Development, Iperboreal Pharma, 65100 Pescara, Italy; t.prosdocimi@iperboreal.com (T.P.); a.arduini@iperboreal.com (A.A.)

**Keywords:** peritoneal dialysis, peritoneum, biocompatibility, peritoneal dialysis solution, ultrafiltration, peritoneal fibrosis, metabolism

## Abstract

Peritoneal dialysis (PD) is a home-based efficacious modality for the replacement of renal function in end-stage kidney failure patients, but it is still under-prescribed. A major limitation is the durability of the dialytic technique. Continuous exposure of the peritoneum to bioincompatible conventional glucose-based solutions is thought to be the main cause of the long-term morpho-functional peritoneal changes that eventually result in ultrafiltration failure. Poor PD solution biocompatibility is primarily related to the high glucose content, which is not only detrimental to the peritoneal membrane but has many potential metabolic side effects. To improve the clinical outcome and prolong the survival of the treatment, PD-related bioincompatibility urgently needs to be overcome. However, combining dialytic and osmotic efficacy with a satisfactory biocompatible profile is proving to be quite difficult. New approaches targeting the composition of the PD solution include the replacement of glucose with other osmotic agents, and the addition of cytoprotective or osmo-metabolic compounds. Other strategies include the infusion of mesenchymal cells or the administration of orally active agents. In the present article, we review the current evidence on efforts to improve the biocompatible and functional performance of PD, focusing on studies performed in vivo (animal models of PD, human subjects on PD).

## 1. Introduction

Peritoneal dialysis (PD) is recognized as an efficacious home-based modality for the replacement of renal function in end-stage kidney failure (ESKF) patients. Depuration from waste products and excess water occurs in PD through exchanges between the peritoneal capillaries and a fluid (PD solution, (PDS) also called PD fluid, or dialysate) infused into the peritoneal cavity via an implanted catheter. After a specified dwell time, the solution is drained out, and fresh dialysate can be re-infused. PDS needs to contain an osmotic agent in order to induce peritoneal ultrafiltration (UF). Standard PDS, used in most PD patients, contains glucose, which is highly osmotic, acceptably safe, rich in calories, and is available at a low cost.

PD is a viable treatment for patients needing chronic dialysis but is still under-prescribed [1]. One prime concern is for the durability of this dialytic technique, since the integrity of the peritoneal membrane (PM) and its capacity to sustain the treatment may decline only a few years after therapy begins [2]. In the short and medium term, infections, primarily from peritonitis and issues with the catheter, are the main factors curbing technique survival [3,4]. In the long term, the non-physiological use of the PM as a dialyzer filter may activate a series of pathologic events such as neoangiogenesis, inflammation, and fibrosis [5]. Such progressive damage to the peritoneum is characterized by a decline in UF capacity, eventually resulting in UF failure, which is the most frequent abnormality in patients on long-term PD and the main cause of PD failure [6].

Several factors can trigger processes harmful to the PM. These include uremia per se [7], mechanical irritation caused by the presence of the intraabdominal catheter [5], and mechanical stretching of the mesothelial cells (MCs) induced by biomechanical cues following introduction into the peritoneal cavity of a large PDS volume [8]. However, the main problem in preserving the functional and anatomical integrity of the PM is the bio-incompatibility of the dialysis fluid [6,9]. Poor PDS biocompatibility is primarily related to the high content of the glucose (molecular weight 180 Da) used to obtain an effective osmotic gradient but that is 10- to 50-fold higher than the serum concentration [10]. Notably, such high-glucose exposure is not only detrimental to the PM (local bioincompatibility) but also has many potential systemic metabolic side effects (systemic bioincompatibility) such as insulin resistance, de novo diabetes, and cardiovascular (CV) disease [11,12]. A recent pilot study [13] showed that steady-concentration PD, performed with the Carry Life UF device (Triomed AB, Lund, Sweden) by adding glucose step-by-step (to maintain the glucose concentration in the PD solution) to a 1.5% glucose-based PD fluid resulted in higher UF rates, and in greater sodium removal compared to a 2.5% dextrose control PD dwell. This novel approach might effectively reduce the amount of glucose in the PD solution, although the extent/magnitude of such a reduction is not clear.

Devising a strategy to reduce/eliminate glucose-associated toxicity without jeopardizing the patient’s health is a major challenge in present-day PD therapy [9]. In particular, coupling osmotic efficacy with a satisfactory biocompatible profile is proving difficult. In the present article, we review the current evidence on efforts to improve the biocompatibility and functional performance of PD, with a focus on studies performed in vivo (animal models of PD, human subjects on PD). 

## 2. Pathophysiology of Peritoneum in Long-Term PD

Long-term changes to the peritoneum in PD, including the mechanisms of peritoneal fibrosis, have been described in recent extensive reviews [5,14,15] and will not be covered in detail here.

The peritoneal membrane consists of mesothelial cells, similar to epithelial cells, adhering to a basement membrane. Below the basement membrane lies the submesothelial layer, which is made up of connective tissue, fibroblasts, and a dense network of blood vessels. During exposure to non-biocompatible PD solutions, the mesothelium undergoes a series of progressive changes, beginning with the loss of microvilli, cellular hypertrophy, and increased vacuolation. Following this, mesothelial cells may detach from the basement membrane, accompanied by thickening of the submesothelial zone. These histologic alterations are linked to changes in the function of the PM, including a gradual rise in solute transport and a reduction in UF capacity. The onset of peritoneal fibrosis, notably, is preceded by functional changes that affect water and solute permeability [16].

Prolonged injury to the mesothelium encourages the conversion of peritoneal mesothelial cells from an epithelial to a mesenchymal state, referred to as either epithelial-to-mesenchymal transition (EMT) or mesothelial-to-mesenchymal transition (MMT). EMT is a prevalent process occurring in physiologic situations, like development and wound healing, as well as in pathologic events such as cancer and organ fibrosis [17].

The loss of epithelial characteristics and the acquisition of mesenchymal features characterizing MMT is partially reversible. During MMT, mesothelial cells lose their cellular polarization, undergo disassembly of the intercellular bonds—such as tight junctions and adherent junctions—and simultaneously transform into a fibroblastic form characterized by increased motility and the ability to generate and release extracellular matrices. Acquiring these new characteristics allows mesothelial cells undergoing MMT to migrate to the sub-mesothelial zone and secrete extracellular matrix, playing a crucial role in fibrogenesis [14].

The loss of cell-to-cell contact in MMT is attributed to the downregulation of epithelial markers such as E-cadherin, cytokeratin, and zonula occludens-1. The downregulation of E-cadherin is a result of the induction of Snail, which directly inhibits E-cadherin transcription. Proteins forming tight junctions, such as claudin and occludin, essential for regulating transport in the peritoneal mesothelium, also undergo expression and localization disturbances in PD patients [14]. Consequently, the integrity of the mesothelial layer barrier is impaired, allowing the dialysate fluid mixture (typically hyperosmotic, acidic, and containing glucose degradation products and advanced glycosylation end products formed in the peritoneal cavity) to come into contact and enter the sub-mesothelium layer, leading to further inflammation [14].

The precise mechanisms driving the EMT in peritoneal MCs remain ambiguous, but it is believed that cytokines, inflammatory factors, and transcription regulators play a role in this process. The existing evidence indicates that EMT in peritoneal fibrosis (PF) is primarily influenced by Smad and non-Smad signaling pathways activated by transforming growth factor (TGF)-β1 [18]. During the early stages of fibrosis, glucose, glucose degradation products (GDPs) and advanced glycosylation end products (AGEs) have been shown to enhance the expression of type I and type II TGF-β receptors in mesothelial cells through the activation of protein kinase C-α [15]. TGF-β1 signalling initiates the phosphorylation of Smad2 and Smad3 via type I TGF-β receptors. Subsequently, Smad2/Smad3 translocate to the nucleus, where they bind directly to DNA, regulating the transcription of target genes associated with fibrosis such as Snail, collagen, alpha-smooth muscle actin (α-SMA), fibronectin, β-catenin, plasminogen activator inhibitor-1, and matrix metalloproteinase-2 [14]. Smad1/5/8 proteins, activated by ALKs in response to bone morphogenetic proteins or other ligands, also translocate to the nucleus to regulate the transcription of target genes [5]. Apart from the Smad-dependent pathway, various non-Smad signaling pathways, including PI3K/Akt, c-Jun N-terminal kinases, Wnt/β-catenin, and ERK/NF-κB, are implicated in the fibrosis process [14].

The presence of high-glucose dialysate leads to the generation of GDPs and the production of AGEs [19]. These substances, as well as glucose itself, have the potential to induce inflammation in the peritoneal cavity by the activation of peritoneal macrophages, subsequently prompting peritoneal MCs to produce cytokines, including interleukin (IL)-1, IL-6, and IL-8. IL-8 and IL-6, initiate the activation of the Janus kinase/signal transducer and activator of the transcription (JAK/STAT) pathway through binding to G-protein-coupled receptors CXCR1/2 or a dimeric cytokine receptor (IL-6Rα and IL-6Rβ/gp130) [20]. This activation triggers loss of the epithelial marker E-cadherin and the deposition of mesenchymal transition markers such as α-SMA, fibronectin, and collagen I. Subsequent activation of the TGF-β1/Smad pathway, a profibrotic pathway, leads to MMT and ultimately results in fibrosis [21].

Long-term exposure to high glucose and the cytokine environment induces macrophage differentiation, with evidence pointing to an increased percentage of M2 macrophages in patients using higher-glucose dialysate, potentially regulated by the Arginase 1 pathway. Macrophage polarization-induced MMT contributes to PF, causing damage to the membrane’s repair and replicative capabilities. A protective response, such as autophagy, is activated to mitigate fibrosis by curbing mitochondrial reactive oxygen species (ROS) production and inflammation. However, excessive autophagy can lead to lysosomal dysfunction and impaired autophagosome clearance, resulting in apoptosis [22]. Chemokines and cytokines released by apoptotic cells further contribute to inflammation and fibrosis progression. In addition to influencing M2 macrophage polarization, high-glucose dialysate inhibits macrophage M1 polarization by activating microsomal prostaglandin E synthase-1 through pyrin domain-containing 3 (NLRP3) inflammation, leading to extracellular matrix component synthesis [23]. Disruption of the balance between extracellular matrix deposition and degradation results in PF, leading to fibrotic tissue development in the abdominal cavity between the injured sides.

Chronic inflammation of the PM induces neoangiogenesis, the formation of new capillaries, increasing the surface area for solute diffusion. Vascular endothelial growth factor (VEGF) plays a crucial role in peritoneal membrane neoangiogenesis. Proinflammatory mediators and cytokines elevated in long-term PD use, such as IL-1b, IL-6, IL-17, and TGF-β, may boost VEGF production. TGF-β, for instance, increases VEGF expression in MCs and fibroblasts, and inhibiting TGF-β has been shown to reduce peritoneal fibrosis and VEGF production in animal experiments. Furthermore, one study demonstrated decreased VEGF levels in patients transitioning from a glucose-based peritoneal dialysis solution to a glucose-free solution (i.e., icodextrin, glycerol, and amino acids), highlighting the role of high-glucose concentrations in upregulating peritoneal VEGF production [14].

## 3. Combating PD-Solution Associated Toxicity

### 3.1. Commercially Available PD Solutions Alternative to Standard Glucose-Based Solutions

Commercially available PD-solution alternatives to standard glucose-based ones are glucose-free dialysates containing icodextrin or amino acids, and also neutral-pH, low-GDP solutions. The main features of commercially available PD solutions are summarized in Table 1.

**Icodextrin** is a colloidal osmotic agent, water-soluble glucose polymer derived from starch, which induces a slow but sustained rate of peritoneal UF [24]. Fluid-handling benefits with icodextrin, such as fewer episodes of fluid overload and improved peritoneal UF, have been described by a recent meta-analysis and systematic review [25]. Evidence of its benefits to glucose metabolism has also been reported in randomized clinical studies [10]. In addition, ESKF patient survival and PD viability may be prolonged by long-term use of an icodextrin-containing PD solution [26]. 

The biocompatibility profile of icodextrin-containing PD solutions is still a controversial issue. The impact on the peritoneal membrane of icodextrin-containing dialysate (the same as for amino acid solutions) could not be evaluated in the biopsies of peritoneal tissues in PD patients [6]. Having a low pH, like the standard PD solution, despite being glucose-free and with a low GDP content, icodextrin dialysate may cause local and systemic inflammation [27]. The development of two chambers, neutral-pH icodextrin dialysate seems indeed to improve the biocompatibility of PD solutions containing icodextrin. In cultured rat MCs, a neutral-pH icodextrin dialysate, unlike conventional acidic icodextrin solution, did not increase the expression of collagen type 1 and 3, alpha-SMA, or P21 mRNA [28]. Moreover, the neutral-pH icodextrin solution was not associated with the stimulation of EMT, the inhibition of cell growth, or the induction of fibrotic changes. These favorable results have been attributed to both the neutral pH and to the GDP content, which is lower than that of the acidic icodextrin dialysate [28], although no clinical evidence is available to further support the above in vitro findings. 

**The amino-acid**-based PD solution (1.1% of amino acids) is the other commercially available glucose-free PD dialysate, with a pH of 6.7 and no GDPs. This approach offers an attractive option in the quest to improve the poor nutritional status of some PD patients. Treatment with a 1.1% amino acid PD solution increased the muscle uptake of amino acids [29]. In a short-term, randomized clinical trial, amino acid-containing dialysate improved the nitrogen balance in eight APD patients [30]. Nutritional parameters may improve with one daily exchange using amino-acid-containing dialysate, but the clinical evidence available is weak [31]. The gluconeogenic potential of amino acids should also be borne in mind when considering that PD patients may already suffer from abnormalities of glucose metabolism. 

The biocompatibility of amino-acid-based PD solution remains uncertain [6]. In a rat PD model, use of an amino-acid-based solution for 12 weeks preserved peritoneal UF and did not increase submesothelial fibrosis, microvascular proliferation, or VEGF expression, unlike standard glucose dialysate [32]. However, other studies have reported the increased generation of nitric oxide, which may have pathophysiologic relevance, in human peritoneal MCs cultured with amino acid PD fluid [33].

Both icodextrin-based and amino-acid-based PD solutions can be used only for a single daily peritoneal exchange and replace up to 50% of the daily glucose load [34]. Two randomized studies showed that, after 6 months of a low-glucose regimen (combination of icodextrin, amino acid, and two glucose-based solutions) in diabetic PD patients, the mean glycated hemoglobin significantly improved as compared to that of a standard glucose solution, with some improvements also being observed for VLDL, triglycerides, and apolipoprotein B [35]. However, the intervention group suffered from a greater number of deaths and adverse events related to the expansion of extracellular fluid volume. Though this might be related to the use of lower concentrations of glucose in the pursuit of better metabolic outcomes, it nevertheless emphasizes the need for efficacious UF with any glucose-sparing strategy.

**Novel neutral- or physiological-pH and low-GDP fluids** using multichambered bags were devised to counteract some components of the standard PD solution that is claimed to be bioincompatible (low-pH, high-GDP content). These PD fluids contain lactate and/or bicarbonate as the pH buffer and are glucose-based. The benefits of neutral-pH, low-GDP solutions, such as preservation of residual kidney function and urine volume, have been demonstrated with convincing evidence in PD patients [36]. The improved preservation of residual kidney function might be related to less-effective fluid removal compared to standard glucose-based dialysate [37]. Indeed, research into the effect of neutral-pH, low-GDP solutions on UF volume as well as on peritoneal transport status has yielded conflicting results [31]. A recent study reported a slow but progressive increase in the peritoneal solute transport rate reaching, after 2 years, levels comparable to those found with standard PD solutions, and thereafter plateauing, while they continue to increase with standard solutions [38]. The Cochrane review [36] indicated (with low evidence) that use of these PD fluids might slightly lower peritoneal UF and increase peritoneal creatinine clearance. On the other hand, no adverse safety signals have been reported concerning any identified harm [31].

Neutral-pH, low-GDP PD solutions are generally being referred to as “biocompatible” PD fluids, supported by many in vitro and experimental in vivo studies [6]. However, recent findings in children on PD suggest that biocompatibility of these solutions cannot be assumed [39]. In a pediatric population, Schaefer et al. [40] examined the effects on PM of low-GDP peritoneal dialysate through repeated biopsies obtained at the time of catheter insertion and after a median time on PD of 13 months. Their results indicate morphological changes, such as an increase in submesothelial thickness (by 20% per year of PD) and of blood microvessel density, accompanied by early peritoneal inflammation, activation of fibroblasts, EMT, and cytokine abundance. Microvascular density showed a correlation with the peritoneal transport rate for small molecules [40]. More recently, Shirai et al. [41] compared the pathologic changes in repeated peritoneal biopsies of children undergoing PD for a median of 3.2 years, exclusively treated with conventional fluids (*n* = 31) or neutral-pH fluids (n = 33). Although the use of neutral-pH fluids induced less severe changes to the PM than conventional fluids, it was nonetheless associated with a detrimental effect. The cumulative dialytic glucose exposure in neutral-pH-treated patients proved to be an independent risk factor for greater thickening of the submesothelial compact zone and the higher density of submesothelial microvessels. Of note, an immunohistochemical study showed a correlation between cumulative dialytic glucose exposure and the expression of VEGF and hypoxia inducible factor (HIF)-1 alpha on the PM of patients using neutral-pH fluids [41]. However, recent findings by repeated biopsies suggest that neutral-pH, low-GDP solutions are protective of peritoneal vascular structures and functions [42]. In adult PD patients treated for more than 5 years, a neutral-pH, low-GDP solution (n = 11) preserved peritoneal endothelial glycocalyx, which affects vascular permeability [43], as compared to conventional fluids (n = 11) [42]. Moreover, Bartosova et al. [44] have shown that, in children on PD therapy with dialysates containing very low or high concentrations of GDPs, omental arterioles exposed to the latter dialysate exhibited a significant increase in AGEs and upregulated genes involved in cell death/apoptosis, along with suppressed genes related to cell viability/survival, cytoskeleton organization, and immune response biofunctions.

There is a need for further studies before we draw any definitive conclusion as to the biocompatibility, and the effects on peritoneal UF and transport status, of neutral-pH, low-GDP solutions.

### 3.2. PD Solutions under Development

The strategies for the development of new solutions for PD are reported in Table 2 and detailed below.

#### 3.2.1. Full Replacement of Glucose with Another Osmotic Agent

The development of effective and safe osmotic agents for PD has been greatly hampered by the risk of hyperosmolar syndrome due to the high solute concentration needed to obtain efficacious peritoneal UF [45].

A possible new substitute for glucose as an osmotic agent in PD solution is **hyperbranched polyglycerol (HPG)**, a biocompatible polymer with a 50–60% dendrimeric structure containing hydroxyl end groups, that is hydrophilic, water-soluble, and chemically stable in aqueous solution [46]. Small HPG polymers (0.5–3 kDa) have been investigated in experimental models as a glucose-sparing strategy for PD treatment. 

In a chronic rat model of PD, the effects over a 3-month period of a glucose-free HPG-based solution were compared to those of glucose-based PDS [47]. While waste removal was similar, the HPG-based solution preserved peritoneal UF significantly better than the glucose-based solution. The experimental solution also induced smaller changes in the structure (thickening of the submesothelial compact zone) and angiogenesis of PM, as well as a lower number of cells expressing VEGF, alpha-SMA, and the macrophage marker MAC387. Transcriptome-based pathway analysis identified the activation of more inflammatory signaling pathways in the PM of rats receiving glucose-based PDS than in the HPG group, including signaling for cytokine production in T cells and macrophages [47]. In a rat model of metabolic syndrome (obese type 2 diabetic ZSF1 rats), La Han et al. [48] reported that, after 3 months of daily intraperitoneal injection use, HPG-containing solution (glucose free) offered better protection of the PM function (peritoneal UF) and structure (thickness, cellular infiltrate) as compared to an icodextrin solution and a low-GDP solution. Moreover, the HPG-based PD solution had fewer systemic adverse effects on the metabolism, serum antioxidant capacity, and immune response [48].

HPG appears to be a promising biocompatible osmotic agent in the development of a glucose-sparing PD solution. However, recent data on the pharmacokinetics of osmotic HPG (1 and 3 kDa) may raise some safety concerns [49]. In this study, rats received, intraperitoneally or intravenously, a single dose of 3H-labelled HPG-containing solutions. Results showed that systemic elimination of the HPG polymers investigated mainly depends on kidney function, implying the risk of HPG accumulation in patients on chronic dialysis [49]. Thus, the metabolism of HPG, and the potential hazards in terms of tissue disposition and plasma accumulation with long-term use, require further studies.

Another osmotic agent under recent development is **Steviol Glycosides (SG)**, active compounds contained in the leaves of the sweetener plant Stevia rebaudiana, which have shown therapeutic effects in several pathologic states [50]. 

Kopytina et al. [51] examined the biocompatibility of SG-containing fluids (which are glucose free) as compared to glucose-based fluids. Dialysis membrane experiments showed that SG has an osmotic capacity similar to that of glucose. In vitro, high-glucose-based PDS induced, in human omental peritoneal MCs, an MMT process with the upregulation of mesenchymal markers (fibronectin, VEGF, Snail 1) and downregulation of E-Cadherin, an epithelial marker. These markers were not significantly affected when cells were exposed to the SG-containing fluid. Concentrations of angiogenic factors VEGF and fibroblast growth factor 2 were also upregulated in supernatants by glucose, but not by SG treatment. Moreover, in a mouse model of PDS exposure for 40 days, treatment with glucose-based PDS induced thickness of PM, an increase in blood vessels, high recruitment of leukocytes, and the release of inflammatory cytokines. In mice exposed to SG-based fluid, all these alterations were markedly reduced, preserving the MC monolayer. The transport capacity of the experimental solution, in terms of urea extracted from the blood, proved to be similar to that of the glucose solution.

These results indicate that SG may be used as an osmotic agent for PDS, to replace glucose, and with a better biocompatibility profile than glucose both in vitro and in vivo. These results, however, are preliminary: the safety and clinical efficacy of SG require further investigation [51], particularly in consideration of the very high daily SG exposures required to achieve an adequate osmotic action for fluid removal and depuration.

#### 3.2.2. Addition of Membrane-Protective Compounds to Glucose-Based PD Solution

One further strategy to improve biocompatibility in PD is the addition of protective compounds in the dialysate containing the standard glucose concentration, so as to counteract peritoneal fluid toxicity. 

The most extensively studied cytoprotective agent is the glutamine-releasing dipeptide, **Alanyl-Glutamine** (Ala-Gln). PD fluids cause cellular stress and suppress the stress-response mechanisms exerted by heat-shock proteins [52], resulting in the increased vulnerability of MCs and altered function of the immunocompetent cells. Supplementation of a PD solution with Ala-Gln improved MC stress response and survival in vitro and ex vivo [53], and reduced peritoneal thickness and angiogenesis and the peritoneal expression of IL-17, alpha-SMA and TGF-beta [54]. Likewise, the addition of Ala-Gln (8 mM) to PD fluid was able to reduce vasculopathy induced by glucose-based dialysate in human umbilical vein endothelial cells [55]. By restoring perturbed cytoprotective responses, Ala-Gln reduced endothelial cell damage and improved cell survival [55]. 

In the first clinical study, a randomized cross-over phase-I/II study, PD effluent samples were collected from 20 PD patients undergoing a peritoneal equilibration test using a standard glucose-based solution with or without Ala-Gln 8 mM 4 weeks apart in a randomized order [56]. The Ala-Gln-supplemented PD solution restored the cellular stress response with a 1.51-fold increase in heat-shock protein expression, and peritoneal glutamine levels. The Ala-Gln addition was likewise associated with improved cellular immune competence. No change was observed in peritoneal UF or small PD solute transport [56].

Samples of PD effluent obtained during that study [56] were then analyzed by metabolomic and proteomic analyses. Targeted metabolomic profiling detected and quantified a higher number of metabolites (198 small molecules) than in previous studies and indicated an anti-oxidative effect by Ala-Gln supplementation [57]. Upregulation of the antioxidant protein thioredoxin reductase-1 might explain the cytoprotective effects of the Ala-Gln additive [58]. A novel proteomic workflow resulted in 2506 unique proteins being identified in PD effluent proteome [59]. Compared to plasma, proteins linked to membrane remodeling and fibrosis were found to be overrepresented in PD effluent, whereas proteins involved in response to stress, host defense, and oxidative stress were underrepresented. After treatment with the Ala-Gln-supplemented dialysate, PD effluent proteomes showed restoration of the biological processes involved in stress response and immune defense, as well as enrichment of cellular processes linked to fibrosis. Improvement of cellular stress response by Ala-Gln addition might occur through the modulation of Akt-dependent pathways [59]. This study confirms the potential of PD effluent proteome in providing a better understanding of the molecular mechanisms involved in ongoing peritoneal pathological processes [60].

More recently, in a randomized crossover study, 50 stable PD patients were treated for 8 weeks with Ala-Gln (8 mM) or placebo added to a neutral-pH low-GDP solution [61]. Ala-Gln supplementation significantly increased the appearance rate of dialysate CA-125 (reflecting improved MC status), and the ex vivo-stimulated release of IL-6, which reflects improved peritoneal immune competence, in PD effluent samples. Ala-Gln-enriched PD fluid was also associated with a reduction in protein loss, and lower levels of biomarkers of systemic inflammation. No significant differences were found between the two dialysates (supplemented or not with Ala-Gln) with respect to peritoneal UF and the transport of some small solutes [61].

A sufficiently powered phase-III trial is now required to assess the impact of Ala-Gln added to PD fluid on hard clinical endpoints [62].

Another potential additive to PDS is **Sulodexide**, a heparinoid formulation comprised of 80% heparin and 20% dermatan sulphate. The supplementation of an overnight glucose-based PD bag with sulodexide (50 mg) for 30 days in 16 CAPD patients improved peritoneal function, as shown by increased urea and creatinine transport and reduced protein loss [63]. These favorable results in peritoneal function were also observed in six CAPD patients who assumed sulodexide orally at monthly increasing dosages for 5 months [64]. No significant change was found in peritoneal UF or peritoneal glucose absorption. The use of sulodexide was associated with a statistically significant and dose-dependent reduction in inflammatory cytokines, such as IL-6, IL-8, and IL-1beta, in the dialysis fluid [64].

More recently, PD effluents were collected from seven CAPD patients after an overnight exchange with 1.5% dextrose, which had sulodexide 0.5 LRU/mL added or not, and the effects on gene expression, secretory activity, and protein synthesis in MCs were examined [65]. The unsupplemented effluent dialysate induced an increased gene expression and the secretion of several molecules, including IL-6, TGF-beta, monocyte chemoattractant protein-1 (MCP-1), VEGF, and vascular cell adhesion molecule 1 (VCAM-1). The use of sulodexide significantly reduced proinflammatory, proangiogenic, and profibrotic phenotypes and the intracellular generation of free radicals. Moreover, supplementation of the PD effluent with sulodexide was associated with a weaker (−21%) stimulation of collagen synthesis [65]. 

The efficiency of sulodexide in the long-term outcome of PD remains to be established.

A novel strategy to protect peritoneal tissue during PD treatment is that of **Molecular Hydrogen** dissolved into PD fluid. The therapeutic application of molecular hydrogen has received increasing attention over the last years, including its use in the treatment of various kidney diseases [66]. Molecular hydrogen is an antioxidant with high biosafety and has demonstrated anti-inflammatory and cell lethality-regulating effects [67].

In a pilot study, Terawaki et al. [68] examined the effects on peritoneal and systemic oxidative stress of a single administration of a dialysate enriched with molecular hydrogen through immersion of the PD bag in H2-rich electrolyzed water for 2 h. Blood and PD effluent samples were obtained from six CAPD patients during a peritoneal equilibration test using standard dialysate or, two weeks later, hydrogen-enriched dialysate. Use of the latter was associated with a reduction in both peritoneal and systemic oxidative stress as measured by the redox state of albumin, without any detrimental effects [68]. In a subsequent clinical trial, six prevalent PD patients were treated for 2 weeks with a molecular hydrogen-dissolved PD fluid [69]. Treatment was well tolerated, with a trend in some patients toward an increase of CA125 and mesothelin in the PD effluent samples, suggesting the enhanced regeneration of MCs.

In an experimental setting, Nakayama et al. [70] examined the effects of molecular-hydrogen-containing dialysate on the PM of experimental PD rats. PD rats treated for 10 days with intraperitoneal injection of hydrogen-rich dialysate, as compared to rats treated with a commercially available low-GDP neutral-pH PD solution, were able to preserve MCs and the PM, with fewer cells in the peritoneal surface tissue testing positive for proliferation, apoptosis and vimentin, together with a dominant presence in the peritoneum of M2 macrophages, which have remodeling/healing actions in damaged tissues [71]. The cytoprotective effect of molecular hydrogen might be exerted through the regulation of phosphatase and tensin homolog (PTEN) activity on anti-fibrotic molecules [72]. After the induction of PF in a mouse model with a high-glucose solution, treatment for 4 weeks with a molecular-hydrogen-rich solution succeeded in alleviating PF as compared to a high-glucose dialysate [73]. This was evidenced by: hematoxylin-eosin staining, Masson trichrome staining, and immunohistochemistry staining for fibronectin, alpha-SMA, and MMP-1 of mouse peritoneum; expression of mesothelial-mesenchymal markers E-cadherin and vimentin; and peritoneal function evaluated by the absorption rate of saline [73]. These results were associated with upregulation of PTEN expression, inhibiting the activation of P13K/AKT/mTOR pathways in peritoneal MCs induced by ROS, a key step in PF [74]. Interestingly, the expression of PTEN was found to be lower in the peritoneum of PD patients with UF requiring catheter removal than in patients without PD at the time of catheter placement [73].

The development of molecular-hydrogen-enriched peritoneal dialysate is a promising approach to attenuating PF, but the specific action mechanisms in cells and the metabolic process of molecular hydrogen in the human body need further investigation.

A recent experimental study investigated the activity of **peroxisome proliferator-activated receptor gamma** (PPARgamma) in modulating the development of PD-induced PF [75]. PPARgamma protein has pleiotropic biological functions; activation of it with specific agents may inhibit fibrosis development in several human tissues [76]. The modulation of renal tubulointerstitial fibrosis by PPARgamma could occur through regulating the expression of glucose transporter type 1 (GLUT1) [77]. Aberrant expressional and functional changes of GLUT1 proved to be implicated in the development of various human fibrotic disorders including PF [78]. Notably, high-glucose levels may significantly alter the expression of GLUT1 in peritoneal MCs [79].

In rat and cellular PF models, Feng et al. [75] demonstrated the fibrosis-regulating role of PPARgamma. In a rat PF model, intraperitoneal injection of PPARgamma agonists (rosiglitazone, 15d-PGJ2) elevated the downregulated expression of PPARgamma and GLUT1 mRNA levels in the PM, decreased blood levels of glucose, creatinine and urea nitrogen, and alleviated PF by reducing the thickness of the submesothelial layer and the collagen fiber content. GLUT1 protein expression proved to be positively regulated by PPARgamma during its modulation of PF progression. The PPARgamma antagonist GW9662 had opposite effects aggravating PF progression. In a cellular PF model, the elevated expression of TGF-beta and alpha-SMA was inhibited by PPARgamma agonists and promoted by GW9662. Moreover, PPARgamma silencing in rat peritoneal MCs markedly decreased the expression of PPARgamma and GLUT1, increased gene expression of TGF-beta and alpha-SMA, and promoted cell proliferation. All these alterations were changed the opposite way by overexpression of PPARgamma [75].

This work discloses new molecular mechanisms underlying functional alterations of peritoneal MCs associated with PF pathogenesis, and new potential targets (PPARgamma expression) for treating PF patients on PD, which deserves further exploration. However, a PPARgamma agonist, rosiglitazone, has been restricted or even withdrawn from the market in most countries owing to concerns about its cardiovascular safety [80].

Another potential PD solution additive is **Melatonin**. Melatonin is a neuroendocrine hormone, secreted by the pineal gland, that upregulates the expression of antioxidant enzymes acting via its receptor and can also inhibit cell apoptosis through the PI3K/Akt/mT0R pathways [81]. 

In different MC types, including peritoneal cells from PD patients after overnight dwelling, melatonin reduced pyroptosis and the downstream triggered proinflammatory responses and neoangiogenesis induced by high glucose [82]. The protective effect of melatonin was confirmed in vivo in mice treated for 6 weeks with a high-glucose dialysate, with or without melatonin injected intraperitoneally. Use of melatonin prevented the increased thickness of PM, maintained peritoneal UF, and reduced glucose absorption. Molecular mechanisms involved in the action of melatonin against MC pyroptosis induced by high glucose include maintenance of mitochondrial integrity, quenching of ROS, and activation of PI3K/Akt/mT0R survival signaling mediated by the melatonin receptor MT1R [82].

Melatonin treatment holds promise in preserving peritoneum integrity in PD, but it requires further exploration.

#### 3.2.3. Addition of Osmo-Metabolic Agents to PD Solution with Low-Glucose Content

The osmo-metabolic approach in formulating new PD solutions is based on the replacement of most glucose in the PD dialysate with other osmolytes [10]. Osmo-metabolites can be defined as those substances possessing both osmotically and metabolically favorable properties. The osmo-metabolic approach might ensure a sort of bioactive glucose sparing, by reducing the intraperitoneal glucose load without compromising UF, and by mitigating the underlying systemic unfavorable metabolic effects induced by the high-glucose load [10]. 

L-carnitine and xylitol are two representative examples of osmo-metabolic agents suitable for use in PD fluid, being chemically stable in aqueous solutions, highly water soluble, and osmotically active. L-carnitine (molecular weight 161.2 Da) is a compound essential for fatty acid metabolism that modulates the levels in the mitochondria of acetyl-CoA, a metabolic intermediate that can affect liver glucose production and muscle glucose disposal [83]. Experimental studies on an L-carnitine-containing PD solution have shown good biocompatibility in several in vitro and in vivo models, while clinical studies in CAPD patients have demonstrated its effectiveness in removing fluid from the peritoneal cavity, as well as better preservation of urine volume and a significant improvement in insulin sensitivity, as compared to glucose-based solutions [84].

In its turn, xylitol (molecular weight 151.2 Da) is a five-carbon sugar alcohol, metabolized within the nonoxidative branch of the pentose monophosphate shunt, giving rise to glycolytic intermediates [85]. A xylitol-containing PD solution proved more biocompatible than a glucose-containing solution in several studies in MCs (Arduini A, Patent PCT/EP2006/060162). In a clinical trial conducted by Bazzato et al. [86], six insulin-dependent diabetic patients on CAPD were treated for a minimum of five months with D-xylitol fully replacing glucose as the osmotic agent in the PD solution. Results showed the maintenance of peritoneal UF and fluid balance, a significant improvement in glycemic control (lowering of glycated hemoglobin, 50% reduction of exogenous insulin dosage) and of the lipid profile [86]. 

Note that in all these clinical studies, the addition to the PD solution of either L-carnitine or xylitol was safe and well-tolerated by patients. 

More recently, we developed a new PD solution, containing both L-carnitine and xylitol, to achieve a favorable synergetic action of the two osmo-metabolic compounds. Low-dose glucose was maintained in the experimental solution to take advantage of its UF ability, at a concentration (27.7 mmol/L) that did not seem to have the deleterious effects of the higher concentration [87]. The biocompatibility of the new PD solution formulation was compared to those of many commercial PD solutions in an experimental model of MCs exposed to the PD fluid only on the apical side, mimicking the condition of a PD exchange [88]. Compelling evidence showed that the experimental PD solution was associated with higher cell viability and better preservation of the integrity of the mesothelial layer (no disruption of the tight junctions triggering EMT and no drop in transepithelial electric resistance). In addition, the L-carnitine+xylitol solutions showed a limited capacity to cause activation of the inflammasome in MCs, allowing for proper cellular homeostasis to be maintained. Analysis of the secretion by MCs of 27 proinflammatory cytokines, growth factors and chemokines, showed a reduced release of TNF-alpha, fibroblast growth factor, and VEGF, compared to conventional PD solutions [88]. Further in vitro data obtained in human mesothelial and endothelial cells support the good biocompatibility of the L-carnitine+xylitol formulation [89]. As compared to a neutral-pH low-GDP solution, the experimental solution was better able to preserve cell viability and membrane integrity, without changes in transepithelial resistance or albumin permeability of MCs, and did not increase the gene expression of TGF-beta or its dependent transcription factor, SNAIL. The activation of mesothelial- and endothelial-mesenchymal transition, as shown by upregulation of alpha-SMA and vimentin and downregulation of E-cadherin both at the gene and at protein levels, was not found in cells exposed to the L-carnitine+xylitol formulation, which also induced a quite mild inflammatory and angiogenic response [89].

Altogether, the available evidence indicates that, unlike available PD fluids, a PD solution containing L-carnitine and xylitol does not exert pro- fibrotic, pro-inflammatory, or pro-angiogenic effects, preserving in the meantime the viability and integrity of mesothelial and endothelial cells. These favorable actions might not be simply attributed to the low-glucose content of the innovative PD solution. Metabolic alterations represent an increasingly recognized pathogenic factor that underlies fibrosis in many organs [90]. Exposure to glucose-based PD fluid led to, in mouse peritoneum, hyperglycolysis, a metabolic alteration accompanied by dynamic mitochondrial changes stimulated by TGF-beta, which is correlated with the development of MMT [91]. Blocking hyperglycolysis by using 2-deoxyglucose, which modulates glycolysis by inhibiting hexokinase 2, inhibited TGF-beta1-induced profibrotic cellular phenotype and peritoneal fibrosis in a mouse model [91]. 

Targeting molecular alterations in MCs, such as metabolic reprogramming (hyperglycolysis), might represent a more appropriate tool to control PD-related PF than targeting fibroblast activation [91]. Supplementation of PD fluids with 2-deoxyglucose demonstrated antifibrotic effects and could improve the functional permeability of mesothelial and endothelial cells of the PM [92]. However, the safety of this approach must be proved in vivo. An alternative strategy is to couple glycolysis with the Krebs cycle by increasing the mitochondrial activity of the pyruvate dehydrogenase complex through reduction of the intramitochondrial acetyl-CoA pool using L-carnitine [14]. As a matter of fact, we recently showed, in a mouse model of PF, that daily intraperitoneal infusion of L-carnitine, xylitol, and low-glucose dialysate for 15 days, compared to a low-GDP solution, prevented the development of fibrosis and did not affect peritoneal angiogenesis (Trepiccione F., oral communication, ERA Congress, Milan, Italy, 2023 [93]). 

A phase-II, prospective, multicenter study to investigate the tolerability and the efficacy of osmo-metabolic agent-based PD solutions in CAPD patients (NCT04001036) was recently concluded [94]. The novel solutions were proven to be well tolerated, and no adverse safety signals were observed. The results of the trial indicate the noninferiority of the osmo-metabolic agent-based PD solutions compared to standard solutions in terms of peritoneal transport and adequacy as targets [94]. 

For the future of PD, it is of the utmost importance to find new osmotic agents targeting its effect on biocompatibility and fluid balance but also, and no less importantly, on the metabolism of the patient. The osmo-metabolic approach makes it possible to reduce the amount of glucose in the PD fluid, and to take advantage of the pharmaco-metabolic properties of the osmolytes to correct potential metabolic abnormalities or deficiencies. The ongoing ELIXIR trial (NCT03994471), a six-month international multicenter randomized study whose primary objective is the noninferiority of the experimental solution based on L-carnitine, xylitol, and low-glucose as compared to a glucose-based PD regarding efficacy and safety, will help to define the role of the novel solution in daily PD clinical practice.

A summary of recent developments in the formulation of novel solutions for PD is shown in Table 3.

### 3.3. Other Approaches

#### 3.3.1. Sodium–Glucose Co-Transport 2 (SGLT2) Inhibition

The use of sodium–glucose co-transport 2 (SGLT2) inhibitors has interesting prospects for PD. These drugs act primarily by inhibiting the coupled reabsorption of glucose and sodium in the proximal renal tubule, leading to diuresis, natriuresis and glycosuria [95]. SGLT2 has been found to be expressed in peritoneal mesothelial cells, and its inhibition has a glucose-lowering effect in the peritoneum [96]. 

Experimental studies indicate the possible benefits of SGLT2 inhibition on the peritoneum.

The intraperitoneal administration of the SGLT2 inhibitor dapagliflozin improved structural and functional peritoneal health [97]. Mice were treated for 5 weeks with high-glucose dialysate without or with the addition of dapagliflozin (1 mg/kg/day). The use of dapagliflozin was associated with a decrease in peritoneal thickening, fibrosis, and microvessel density as compared to mice treated with unsupplemented dialysate. Moreover, peritoneal UF improved without the development of a high-glucose transport status. Intraperitoneal addition of dapagliflozin also inhibited the SGLT2 transcriptional upregulation induced by glucose-only PD solution [97]. Use of intraperitoneal canagliflozin (10 mg/kg/day) in a PD rat model of chronic high-glucose exposure prevented PM thickening, reduced the deposition of fibrotic proteins (fibronectin, alpha-SMA, and COL1A2), and inhibited the HIF-1 alpha/TGF-beta/p-Smad3 signal pathway, consistent with results in human peritoneal MCs [98]. Peritoneal function also was improved, as shown by increased UF, reduced glucose uptake, and decreased transport of creatinine. These favorable results may be related to improvement of hypoxia and inhibition of HIF-1 alpha and TGF-beta/p-Smad3 signaling pathways brought about by canagliflozin [98], since both hypoxia and HIF-1alpha participate in the development of PF [99,100].

Other authors reported in a mouse PD model, that intraperitoneal administration of SGLT2 inhibitor empagliflozin (6 mg/kg/day) for four weeks ameliorated the features of PF induced by high-glucose PDS such as increased peritoneal thickness and permeability and upregulation of collagen and alpha-SMA expression [101]. Inflammatory cytokines including TNF-alpha and IL-6, and TGF-beta/Smad signaling pathways, also decreased in the empagliflozin-treated group. Empagliflozin also reduced the functional impairments of the PM in terms of absorption rate of glucose from the dialysate and transport rate of blood urea from the plasma [101]. These favorable results were not confirmed by Martus et al. [102], who reported no effects from empagliflozin (given as an intravenous infusion) on peritoneal glucose uptake or UF. That study however examined a 120-min experimental model in rats with normal renal function, which may at least partly explain the conflicting evidence. More recently, empagliflozin administered orally by gavage at a daily dose of 3 mg/kg to PD rats for four weeks showed a protective effect against high-glucose-based PDS-induced EMT (excessive collagen deposition, decreased expression of E-cadherin, increased expression of collagen I, fibronectin, and alpha-SMA), and suppressed oxidative stress [103]. These effects occurred through activation of the nuclear factor erythroid-2-related factor 2 (Nrf2)/heme oxigenase-1 (HV-I) signaling pathway. Peritoneal UF volume and glucose absorption also significantly improved in the PD group treated with empagliflozin-added dialysate as compared to untreated animals [103].

Recent preliminary studies have examined the clinical application of dapagliflozin in PD patients. Fifty type-2 diabetic patients on automated PD received oral dapagliflozin 10 mg daily for 6 months [104]. Treatment was associated with a significant improvement in urine volume, peritoneal UF, and mean systolic blood pressure. Peritoneal transport status did not change. Significant improvements in fasting blood sugar and glycated hemoglobin with reduced insulin requirement were also observed [104]. Other authors evaluated the effects of oral dapagliflozin 5 mg daily in four incident PD patients (3 of them diabetic) with fluid overload [105]. Dapagliflozin increased peritoneal UF, allowing a euvolemic status to be achieved after 14 (three patients) or 28 days, and was then stopped. Urine volume did not change; no episodes of hypoglycemia or hypotension were observed [105]. A very recent one-month retrospective pilot study evaluated the effects of oral dapagliflozin (10 mg daily) on the function of PM in 20 CAPD patients [106]. Administration of dapagliflozin did not significantly reduce the absorption of glucose across the PM, which proved decreased in 13 patients and increased in seven. Peritoneal UF volume did not change. Dapagliflozin use was also associated with a reduction in systemic IL-6 levels and in peritoneal VEGF, which might result in PM protection but was again not statistically significant, possibly due to the small sample size [106]. 

SGLT2 inhibitors demonstrated potential kidney and CV benefits in CKD patients [107]. Evidence from experimental studies and post hoc analyses indicate that SGLT2 inhibitors may be effective in preventing CV and mortality outcome in patients on dialysis also [95]. Potential benefits of SGLT2 inhibition in PD patients include slowed decline of residual kidney function, protection of the peritoneal membrane, and CV effects [108]. There is accumulating rationale to support use of SGLT2 inhibitors in patients on PD, though their safety and efficacy in this patient population remains to be ascertained in adequately powered clinical trials [95].

#### 3.3.2. Dipeptidyl Peptidase 4 Inhibition

Some studies focused on the use of dipeptidyl peptidase 4 (DPP4) inhibitors, glucose-lowering agents that act by prolonging the action of incretins, resulting in increased insulin release and decreased glucagon production [109]. DPP4 inhibitors may also exert anti-inflammatory, anti-apoptotic, and anti-fibrotic effects [110].

DPP4 is expressed in human MCs, and its expression is increased in PF [111]. Studies in mice showed that the DPP4 inhibitor linagliptin, administered by once-daily oral gavage (10 mg/kg), ameliorated PF induced by methylglyoxal, suppressing the receptor expression of glucagon-like peptide 1 [112]. In DPP4-deficient rats, DPP4 activity in MCs proved to be significantly correlated with the functional impairment of PM; treatment with oral sitagliptin (600 mg/kg) for 20 days improved peritoneal functional parameters and preserved the peritoneum with protection from PF [111]. 

Interestingly, a retrospective analysis from the Taiwan National Health Insurance Renal Database indicated that the incidence of transition from PD to hemodialysis due to PD failure was significantly lower in diabetic patients treated with DPP4 inhibitors [111]. 

More recently, Jo et al. [113] reported, in a chronic infusion model of rat PD, that oral administration of sitagliptin (5 mg/kg/day) for 8 weeks ameliorated PF and significantly improved both peritoneal UF and solute diffusion as compared to rats without sitagliptin treatment. The effects were associated with improvements in the protein expression of claudin-1 and claudin-15 in peritoneal MCs as compared to PD rats that were not supplemented. Claudin-1 and claudin-15 are tight-junction proteins that determine paracellular ion permeability and selectivity [114]. In cultured human peritoneal MCs, sitagliptin co-treatment reversed the altered expression of claudin-1 and claudin-15 proteins, as well as the associated changes in paracellular permeability induced by the PF-inducer TGF-beta1 [113].

The protective effects of DPP4 inhibitors in PD need to be confirmed in clinical trials.

#### 3.3.3. Stem Cells

Stem cells (SCs) can differentiate into any cell of the human body and are responsible for the development and regeneration of organs and tissues. SCs may influence cellular differentiation and exert immune regulation and cellular repair effects [115], and their therapeutic potential has been investigated in several pathologies including peritoneal inflammation and fibrosis, mesenchymal SCs (MSCs) being the most extensively studied [116]. In vitro and in vivo studies showed that MSCs might represent a novel and effective treatment of PF, mainly through paracrine activity to accelerate healing or differentiation into functional repair cells [115]. In a uremic rat PF model, intravenous injection of adipose-derived MSCs displayed anti-inflammatory (avoidance of leukocyte infiltration and overexpression of inflammatory cytokines including IL-1, IL-6, and TNF-alpha) and anti-fibrotic (regulation of TGF-beta, collagen, and fibronectin) effects in the PM [117]. Yang et al. [118] demonstrated that both adipose-derived and bone marrow-derived MSCs attenuated PM thickening in a rat model of dialysis-induced PF. These therapeutic effects were associated with M2 macrophage polarization (a phenotype exerting anti-inflammatory properties) promoted by IL- 6 secreted by MSCs [118]. Treatment with a single injection into the peritoneal cavity of umbilical cord-derived MSCs prevented UF loss and reduced the damage to PM in terms of mesothelial thickness, angiogenesis, inflammation and fibrosis in a rat model of PF [119]. Similar effectiveness in the protection of PM from PD solution-induced injury and dysfunction was observed in a rat model of biologically incompatible PDS induction upon treatment with PD effluent-derived MSCs [120]. MSCs can indeed be obtained from discarded PD effluent [121], which may provide a practical unlimited source of MSCs [122]. It has to be noted that in preclinical studies the improved PF by use of SCs was associated with an improvement in peritoneal permeability function, as assessed by peritoneal UF, glucose transport, and solute permeability [116]. 

Recent clinical studies in PD patients showed the safety and feasibility of MSC infusion. In a prospective, non-randomized phase I study, 10 patients on PD received an intravenous infusion of autologous adipose-derived MSCs and were followed up for 6 months [123]. Treatment was well tolerated, and no major safety signals were observed. Peritoneal UF significantly increased by 20.5%, while parameters of dialysis efficiency did not change, though a potential positive change in solute transport was found [123]. Jiang et al. [124] followed up for four years 24 CAPD patients who received umbilical cord-derived MSC treatment injected intravenously (n = 20) or directly into the renal artery. Treatment with MSCs was associated with a significant increase in erythropoietin and hemoglobin levels and the Activities of Daily Score, and with a significant decrease of high-sensitivity C-reactive protein and cystatin C levels. Urine volume was improved, while parameters of dialysis adequacy were stable [124]. 

SC therapy has great potential as a new strategy for alleviating PF. However, approaches for treatment are not yet standardized and there is no consensus as to the transplantation protocol [115]. Thus, further research is needed to define the application of SC therapies in the clinical practice of PD.

#### 3.3.4. Indobufen

In a prospective randomized study, Liu et al. [125] evaluated the effects of indobufen on the microinflammatory state and peritoneal transport function in PD patients. Indobufen is an antiplatelet drug with anticoagulant, antithrombotic, and anti-inflammatory effects [126]. Sixty CAPD patients were randomized to the control and oral indobufen (100 mg twice daily) groups. After six months, microinflammatory parameters (platelet-to-lymphocyte ratio and TNF-alpha, in both serum and peritoneal effluent) were significantly decreased in the indobufen group compared to before the start of treatment and to the control group at month 6, suggesting that indobufen reduced both systemic and local microinflammation. Treatment with indobufen was also associated, at 6 months, with a significant decrease in both serum and effluent dialysate of PF indexes such as TGF-beta and cell fibronectin. Moreover, peritoneal transport function was improved in the indobufen group, which may be related to the improvement of microinflammation and PF. Indobufen use showed a favorable safety profile [125]. Long-term data are now warranted for this approach.

#### 3.3.5. Roxadustat

Roxadustat is an oral inhibitor of HIF-prolyl hydroxylase that improves renal anemia by stimulating erythropoietin gene transcription, thereby increasing endogenous erythropoietin levels, and improving iron homeostasis [127]. Roxadustat acts by stabilizing HIF-alpha, which then accumulates in the cell and drives the transcription of hundreds of genes with various biological roles [128]. It has been shown that roxadustat may have anti-inflammatory effects, which could be of benefit in preserving organ function and structure, while also delaying the progression of fibrosis [129].

Wang et al. [130] very recently investigated the potential protective role of roxadustat against PD-induced PF. In a PD fibrosis-induced mouse model, roxadustat administration by oral gavage (10 mg/kg and 20 mg/kg) for 4 weeks ameliorated the morphological features of PF and peritoneal permeability, reduced the levels of inflammatory cytokines IL-6 and TNF-alpha, and inhibited the EMT process, as shown by the increased expression of E-cadherin and reduced expression of fibronectin, alpha-SMA, and collagen I. These results were confirmed in vitro. The protective effect of roxadustat could involve the TGF-beta/Smad signaling pathway [130].

These results are interesting but require validation. HIF stimulation, as stated above, seems to be at odds with respect to the inhibition of fibrosis-driven processes or even with respect to epithelial/mesothelial-mesenchymal transition, as triggered by glucose in MCs in PD patients [91]. Indeed, hypoxia/HIF-1 alpha is one of the major events regulating different EMT transcriptional regulators, including Snail, Twist1, ZEB1, ZEB2, and SIP1 [131,132].

#### 3.3.6. *Lactobacillus casei* Zhang

Gut microbiota can regulate the biological activities of remote organs through the modulation of production and dissemination of several bioactive metabolites [133]. Increasing evidence shows that dysregulated microbiota accompanies the onset and the progression of various diseases [134]. In particular, gut dysbiosis is directly involved in the genesis of inflammatory and fibrotic processes in various organs, and its correction by prebiotics and probiotics was proven to be clinically effective in mitigating fibrotic alterations [135].

Gut dysbiosis is prevalent in patients on PD, due to renal failure and PD itself [136]. Zhongcai et al. examined the effects of the probiotic strain *Lactobacillus casei* Zhang in a mouse model of PD-induced PF [137]. *Lactobacillus casei* Zhang has antioxidant and anti-inflammation properties, which could mitigate kidney injury and fibrosis [135]. Oral gavage administration of *Lactobacillus casei* Zhang (1 × 10^9^ CFU) for 6 weeks significantly attenuated high-glucose PDS-induced PF, as was evidenced by reduced submesothelial thickness and collagen deposition, and by UF tests. Macrophage infiltration and inflammatory cytokines were effectively reduced in PD effluent. Moreover, *Lactobacillus casei* Zhang administration corrected the gut microbiota profile, enriching the beneficial bacteria that produce short-chain fatty acids. The therapeutic effects of *Lactobacillus casei* Zhang may stem from the induced elevated levels of butyrate, leading to suppression of Nf-kB-mediated inflammation by upregulating PPARgamma [137].

#### 3.3.7. Tamoxifen

Other studies investigated the anti-fibrotic role of tamoxifen, a selective estrogen receptor 1 modulator that can effectively inhibit MMT and neoangiogenetic processes [138,139].

In a mouse model of PD-induced PF, treatment with tamoxifen citrate 10 mg/kg/day by oral gavage mitigated PF induced by high glucose (peritoneum thickness, collagen deposition), improved peritoneal UF, and decreased the peritoneal solute transport rate [140]. In peritoneal cells upon high-glucose stimulation, tamoxifen was able to preserve cell viability and morphology, and suppressed changes to MMT marker proteins. The protective role exerted by tamoxifen against fibrosis proved to be the blocking of translocation into the nucleus of estrogen receptor 1 to transcribe H19, a long noncoding RNA that promotes fibrosis by activating the proangiogenic cytokine VEGFA.

Interestingly, in peritoneal tissue from six PD patients at the time of catheter removal due to UF failure (PD duration > 5 years), high expression of estrogen receptor 1 was found in the nucleus of MC, as well as a positive correlation between estrogen receptor 1 (or H19) RNA levels and high peritoneal solute transfer rate [140]. 

Strategies to improve the outcome of PD indirectly targeting the composition of PD solution are summarized in Table 4.

#### 3.3.8. Disposable Ultra-Fine Endoscope

Recently, a disposable ultra-fine endoscope designed specifically for use in PD patients was developed [141]. This endoscope can be inserted into the PD catheter, allowing for non-invasive visualization of the intraluminal side of the catheter and the PM surface. Results obtained from a pre-clinical study in pigs and from 10 patients on PD therapy demonstrated use of the device to be safe and able to provide detailed images. Since this novel diagnostic approach can be used repeatedly, it might reveal temporal changes in the peritoneal status and provide further insights on the pathophysiology of the peritoneum during the course of PD therapy [141].

## 4. Conclusions

In PD therapy, there is an urgent need to ameliorate the biocompatibility of the treatment to improve the long-term results of this technique on ESRD patients. Since some unfavorable factors are not modifiable, efforts have focused mainly on the alleged principal pathogenic cause of PD bioincompatibility, the high-glucose concentration of the PD solution and the related consequences. However, the search for an ideal PD solution remains elusive [31], and glucose-based PD fluids still predominate. The current evidence for novel PD solutions under development is limited to experimental or short-term clinical studies. Nevertheless, some new approaches appear promising for clinical applications. Strategies indirectly targeting the composition of PD solution are also being exploited, with most investigating drugs that are active via oral administration. Clearly, all experimental studies need validation in vivo, just as initial clinical studies need longer safety and efficacy assessments. Hopefully, we will soon have a more biocompatible PD therapy, for the benefit of the patient. 

## Figures and Tables

**Table 1:** Main features of commercially available solutions for peritoneal dialysis.

	pH	Low GDPs	Glucose Load	Glucose Sparing	Biopsies of Peritoneum	Potential Systemic Advantage	Potential Peritoneal Advantage
Glucose-based	5.2–5.5	NO	YES	NO	YES	Nutritional	Osmotic
Biocompatible lactate and/or bicarbonate buffer	7.0–7.4	YES	YES	NO	YES	Nutritional	Osmotic and pH
Icodextrin	5–6	YES	NO	YES	NA	Volume control	Long-dwell UF
Aminoacids	6.6	NA	NO	YES	NA	Protein synthesis	Osmotic

GDPs, glucose degradation products; NA, not available; UF, ultrafiltration.

**Table 2:** Strategies for the development of new solutions for peritoneal dialysis.

Full replacement of glucose with another osmotic agent Addition of cytoprotective compounds to PD solution with standard glucose content Addition of osmo-metabolic agents to PD solution with low-glucose content

**Table 3:** Summary of recent developments in the formulation of novel solutions for PD.

Approach	Agent	Clinical Stage	Treatment Duration	Biocom-Patibility	Peritoneal UF	Open Issues
Full glucose replacement	Hyperbranched polyglycerol	Pre-clinical (in vivo rat models)	3 months	↑	Preserved	Risk of accumulation in ESKF patients
Full glucose replacement	Steviol glycosides	Pre-clinical (in vivo mouse model)	40 days	↑	NA	Safety of the needed very high daily exposure
Addition of cytoprotective compounds	Alanyl-glutamine	Clinical trial (CAPD, APD)	8 weeks	↑	Comparable to glucose	Need for a phase III study
Addition of cytoprotective compounds	Sulodexide	Clinical trial (CAPD)	30 days	↑	No change	Efficacy and safety in the long term
Addition of cytoprotective compounds	Molecular hydrogen	Clinical trial (CAPD)	2 weeks	↑	NA	Action mechanisms in cells and metabolic process in the human body
Addition of cytoprotective compounds	PPARgamma agonists	Pre-clinical (in vivo rat model)	4 days	↑	NA	Efficacy and safety in humans
Addition of cytoprotective compounds	Melatonin	Pre-clinical (in vivo mouse model)	6 weeks	↑	Preserved	Efficacy and safety in humans
Addition of osmo-metabolic compounds	L-carnitine and xylitol	Clinical trial (CAPD)	4 weeks	↑	Comparable to glucose	Efficacy and safety in the long term

PD, peritoneal dialysis; ↑, improvement as compared to control PD solution; UF, ultrafiltration; ESKF, end-stage kidney failure; NA, not available; CAPD, continuous ambulatory peritoneal dialysis; APD, automated peritoneal dialysis.

**Table 4:** Summary of exploiting strategies to improve the outcome of PD indirectly targeting the composition of PD solution.

Agent	Clinical Stage	Administration, Dosage	Treatment Duration	Biocom-Patibility	Peritoneal UF	Open Issues
Dapagliflozin	Clinical trial (APD)	Oral, 10 mg/day	6 months	↑	Improved	Efficacy and safety in the long-term
Canagliflozin	Pre-clinical (in vivo rat model)	Intraperitoneal, 10 mg/kg/day	5 weeks	↑	Improved	Efficacy and safety in humans
Empagliflozin	Pre-clinical (in vivo mouse model)	Intraperitoneal, 6 mg/kg/day	4 weeks	↑	NA	Efficacy and safety in humans
Linagliptin	Pre-clinical (in vivo mouse model)	Oral gavage, 10 mg/kg/day	6 weeks	↑	NA	Efficacy and safety in humans
Sitagliptin	Pre-clinical (in vivo rat model)	Oral gavage, 5 mg/kg/day	8 weeks	↑	Improved	Efficacy and safety in humans
Stem cells	Clinical trial (CAPD)	Intravenous infusion of autologous adipose-derived MSCs	6 months	↑	Improved	Need for standardized treatment approach and transplantation protocol
Indobufen	Clinical trial (CAPD)	Oral, 100 mg twice daily	6 months	↑	NA	Efficacy and safety in the long-term
Roxadustat	Pre-clinical (in vivo mouse model)	Oral gavage, 10 and 20 mg/kg	4 weeks	↑	Improved	Efficacy and safety in humans
*Lactobacillus casei* Zhang	Pre-clinical (in vivo mouse model)	Oral gavage, 1 × 10^9^ CFU	6 weeks	↑	Preserved	Efficacy and safety in humans
Tamoxifen	Pre-clinical (in vivo mouse model)	Oral gavage, 10 mg/kg/day	15 days	↑	Improved	Efficacy and safety in humans

PD, peritoneal dialysis; UF, ultrafiltration; APD, automated peritoneal dialysis; ↑, improvement as compared to control PD solution; NA, not available; CAPD, continuous ambulatory peritoneal dialysis; MSCs, mesenchymal stem cells; CFU, colony forming units.

## Data Availability

No new data were created or analyzed in this study. Data sharing is not applicable to this article.
